# Exploring the Relationship between Blood Flux Signals and HRV following Different Thermal Stimulations using Complexity Analysis

**DOI:** 10.1038/s41598-018-27374-5

**Published:** 2018-06-12

**Authors:** Guangjun Wang, Shuyong Jia, Hongyan Li, Ze Wang, Weibo Zhang

**Affiliations:** Institute of Acupuncture and Moxibustion, China Academy of Chinese Medical Sciences, Beijing, China

## Abstract

To investigate the relationship between local blood flux and heart rate variability following different thermal stimulations, healthy subjects were recruited and subject to different thermal stimulations on the right forearm. Multiscale entropy and multiscale fuzzy entropy were used to measure the complexity of the local blood flux, and the approximate entropy was calculated to evaluate the HRV complexity. The results indicated that thermal stimulation significantly increased local blood flux and that different temperature stimulations resulted in different complexities in local blood flux. A 42 °C or 44 °C thermal stimulation, other than stimulations below 42 °C, resulted in a moderate correlation between local blood flux and heart rate variability complexity. The results provide a new perspective in terms of complexity to explore the relationship between skin blood flux signals and cardiac function.

## Introduction

Moxibustion is an important component of Traditional Chinese Medicine (TCM) theory and practice. It is an external therapy using a burning moxa stick to produce a warm sensation at certain acupoints^1^. Unlike mechanical stimulation of acupuncture, thermal stimulation is the key point, and temperature plays an essential role during moxibustion^1,2^. Previous studies have shown that different temperatures might produce different efficacies^2,3^, but the exact mechanism is not clear. No matter how complex the cascade reaction following thermal stimulation, local blood flux is among the most direct non-invasive indicators to investigate the effect mechanism, and a previous study has shown that local temperature is an essential factor regulating skin blood flux^4^. Therefore, deep analysis of blood flux signals is among the effective means to explore the mechanism of thermal stimulation^5,6^. On the other hand, acupuncture-related stimulation has been suggested to treat disorders by regulating the body’s autonomic functions^7^, and heart rate variability (HRV) analysis is a useful tool to monitor these autonomic functions^8^. Thus, HRV analysis is typically used to evaluate acupuncture effects^9–11^. Previous results have indicated that thermal stress can significantly affect heart rate variability^12^. Considering the complex control mechanisms of the heart, it is reasonable to assume that nonlinear dynamics are involved in the regulation of HRV. Recently, studies have indicated that nonlinear analysis of heart dynamics may provide more powerful information than time- or frequency-domain results of heart rate variability^13^. As a meaningful index, Approximate Entropy (ApEn) could be used to differentiate healthy subjects from patients with coronary artery disease^14^, and as a case, it can be used to measure the complexity of HRV^15^.

It is usually considered that skin blood perfusion (SkBF) is composed of multiple components, and thus, spectrum analysis provides a non-invasive method to examine the regulating mechanisms of skin blood flux^5,16^. It has been established that the cardiovascular system is a complex nonlinear dynamical system and that skin blood perfusion regulation is also a nonlinear process^17^. From this perspective, spectrum analysis of laser Doppler flowmetry (LDF) is unable to characterize the more dynamical properties of blood flux oscillations^17^. During recent years, many studies have indicated that nonlinear dynamic analysis could be a candidate method to study SkBF response activity^18^, and sample entropy (S_E_) has been used both to discriminate different microcirculatory responses in stroke patients^19^ and evaluate the SkBF response resulting from thermal stresses^20^.

Compared to sample entropy, multiscale entropy analysis has an advantage and provides a more powerful tool for complexity measurement^21,22^. It has been successfully utilized to distinguish healthy status from disorder conditions^23^. To measure vascular dynamics, multiscale entropy has also been used to analyse laser Doppler flowmetry time series^24,25^. Considering that multiscale fuzzy entropy has a more significant correlation with multiscale sample entropy than that of other multiscale entropies^26^, both sample entropy and fuzzy entropy at different scales were analysed in the current study.

In summary, thermal stimulation can affect not only local blood flux but also heart rate variability. However, the relationship between local blood flux and heart rate variability has not received attention in terms of complexity. Therefore, in this study, multiscale entropy analysis and multiscale fuzzy entropy were calculated to evaluate the complexity of local blood flux signals. Furthermore, the relationship between the complexity of the local blood flux signal and HRV was analysed.

## Results

**Figure 1:**
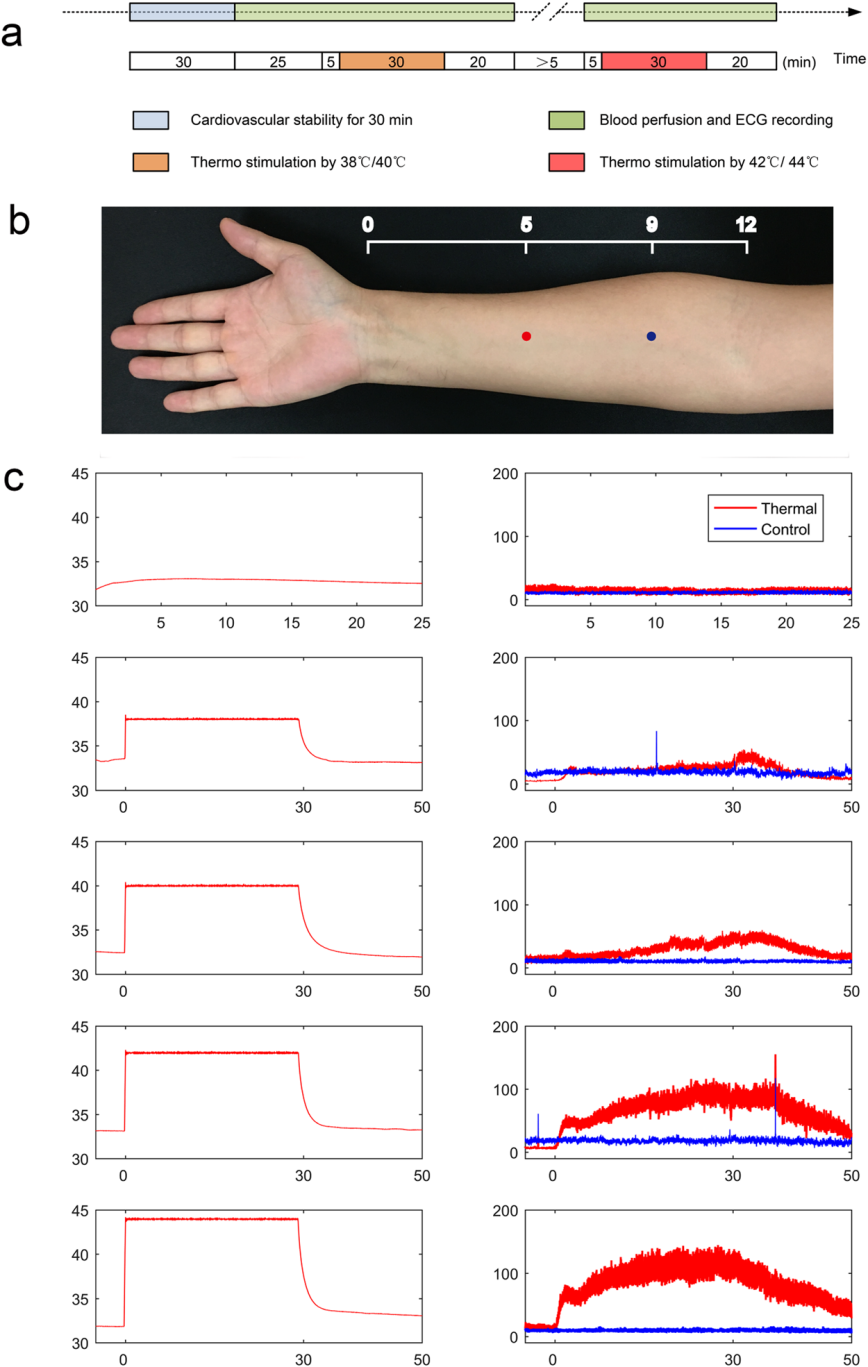
Experimental design and case subject raw data for blood perfusion flux. (**a**) Experimental design. (**b**) Thermal stimulation point (red) and control point (blue) location on the right forearm. The thermal stimulation point is on the anterior aspect of the forearm, between the tendons of the palmaris longus and the flexor carpi radials, 5 B-cun proximal to the palmar wrist crease. The control point is 7 B-cun proximal to the palmar wrist creas^51^. (**c**) Temperature of the stimulation point (left) and the blood perfusion signal at both the stimulation and control points (right).

A total of 60 subjects were included in the current study. Detailed information on the subjects receiving thermal stimulation (n = 30) is summarized in Table 1. The experimental design and blood flux recording for the forearm are shown in Fig. 1a. The recording positions of both the thermal stimulation and control points are shown in Fig. 1b. The different temperature stimulation conditions and related blood flux signals of the individuals are shown in Fig. 1c. Detailed information on the parallel blank control group (n = 30) is summarized in Table S1. The experimental design and case results of the parallel blank control are shown in Fig. S1.

**Table 1:** Subject characteristic descriptions (8M/22 F).

	Min	Max	Mean ± SD
Age (years)	22	48	27 ± 5
Height (cm)	155	178	165.47 ± 6.84
Weight (kg)	45	95	59.53 ± 11.81
BMI	17.56	30.32	21.64 ± 3.29

**Figure 2:**
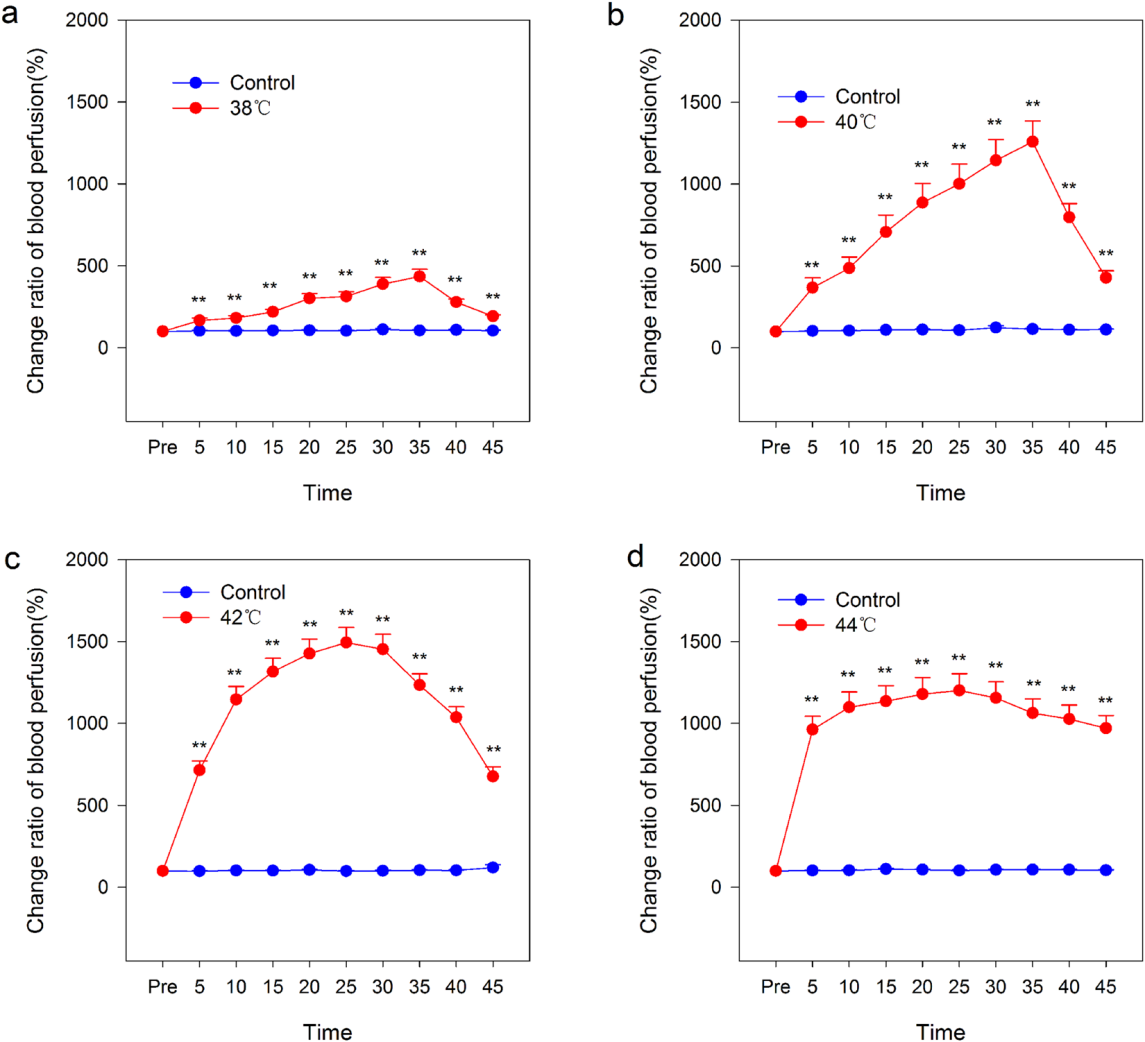
Blood perfusion flux signals at both the stimulation and control points. (**a**) 38 °C thermal stimulation. (**b**) 40 °C thermal stimulation. (**c**) 42 °C thermal stimulation. (d) 44 °C thermal stimulation. **P < 0.01, paired-t test (n = 30).

The results of this study suggest that no matter the temperature of the stimulation, blood flux significantly increased after 5 min of stimulation. However, under different temperatures of stimuli, the patterns of the blood flux signal were completely different. Under stimulations of 38 °C (Fig. 2a) and 40 °C (Fig. 2b), blood flux increased linearly with stimulation prolongation, and the difference is the increased slope of the blood flux signal. Five minutes after stimulation cessation, the blood flux reduction process also showed linearity. Under a stimulation of 42 °C (Fig. 2c), the blood flux increased first and then decreased in a parabolic pattern. However, with 5 min of 44 °C (Fig. 2d) stimulation, the blood flux was near the maximum extremum. In the parallel blank control group, the blood flux showed no changes during each epoch (Fig. S2a and b).

**Figure 3:**
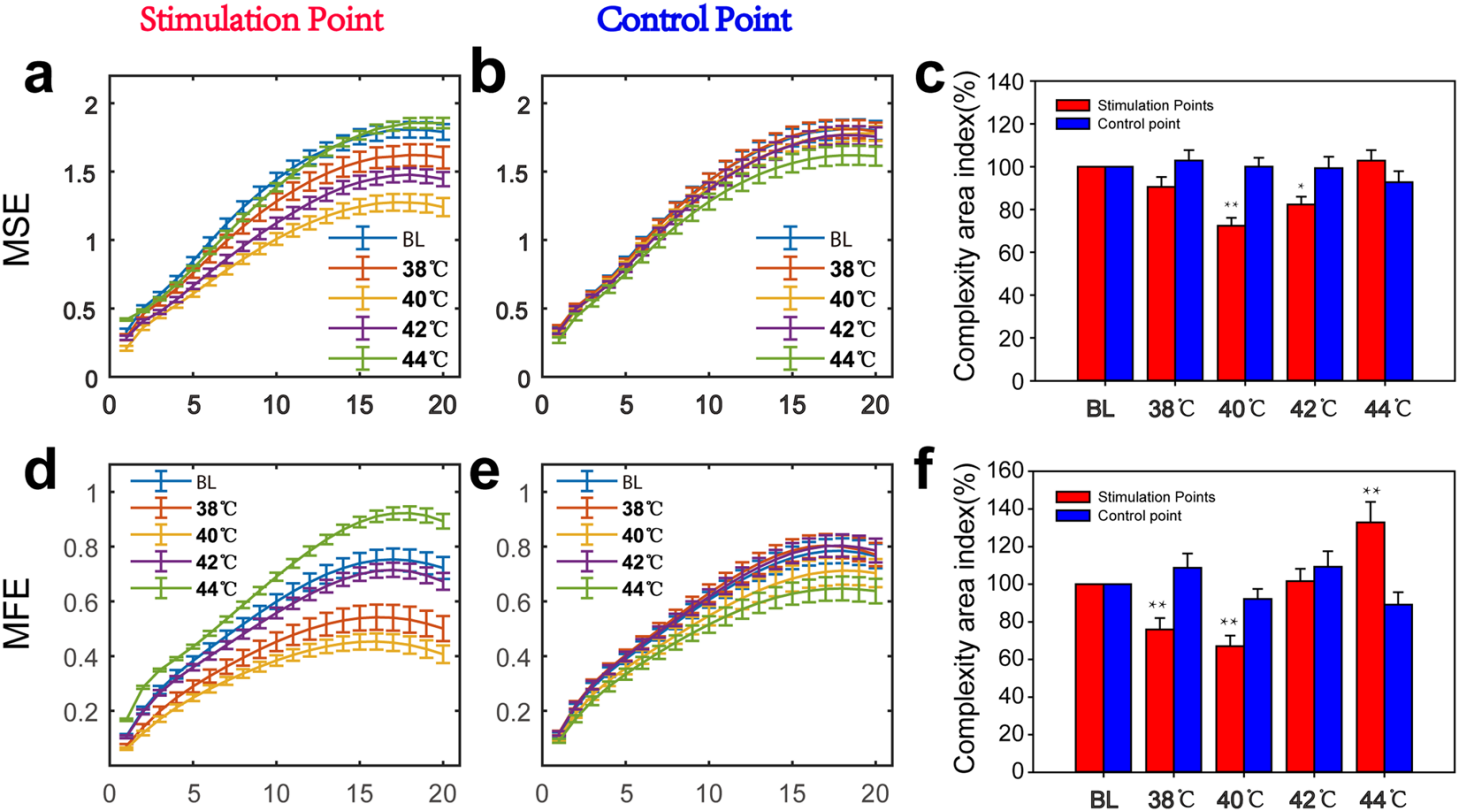
Complexity of the blood perfusion flux signals. (**a**) MSE result of different scales at the stimulation point. (**b**) MSE result of different scales at the control point. (**c**) Complexity area index of different conditions and different points. The complexity area index was obtained as the area under the multiscale entropy curve of Fig. 3a,b. (**d**) MFE result of different scales at the stimulation point. (**e**) MFE result of different scales at the control point. (**f**) The complexity area index of different conditions and different points. The complexity area index was obtained as the area under the multi-scale entropy curve of Fig. 3d,e. *P < 0.05; **P < 0.01, paired-t test (n = 30). MSE, multi-scale entropy. MFE, multiscale fuzzy entropy. BL, baseline condition without stimulation. Data are presented as the mean ± SE.

**Figure 4:**
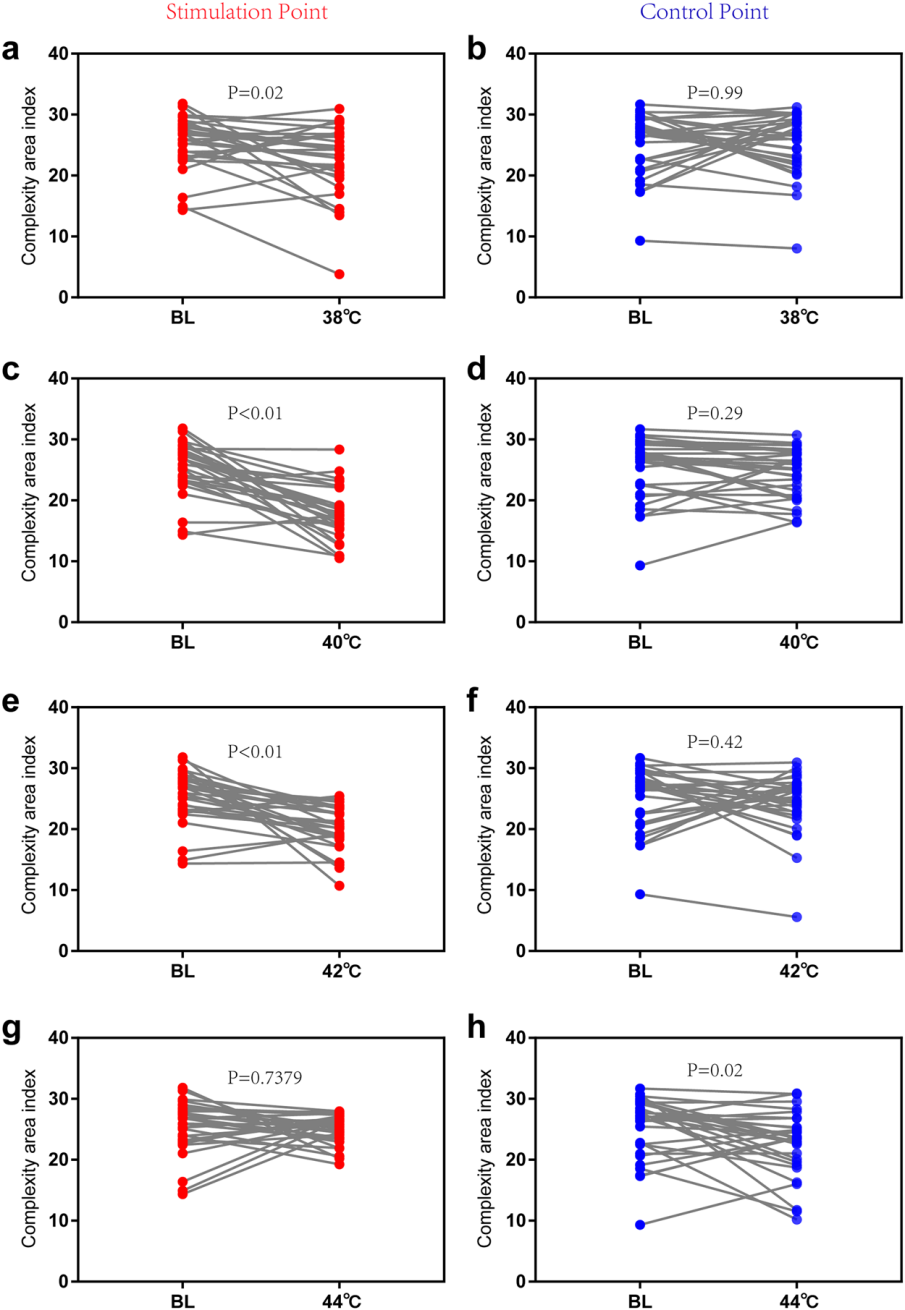
Contrast in MSE between baseline and other thermal stimulation conditions. (**a**) Stimulation point with 38 °C stimulation. (**b**) Control point with 38 °C stimulation. (**c**) Stimulation point with 40 °C stimulation. (**d**) Control point with 40 °C stimulation. (**e**) Stimulation point with 42 °C stimulation. (**f**) Control point with 42 °C stimulation. (**g**) Stimulation point with 44 °C stimulation. (**h**) Control point with 44 °C stimulation. Paired-t test (n = 30). Data are presented as the mean ± SE.

**Figure 5:**
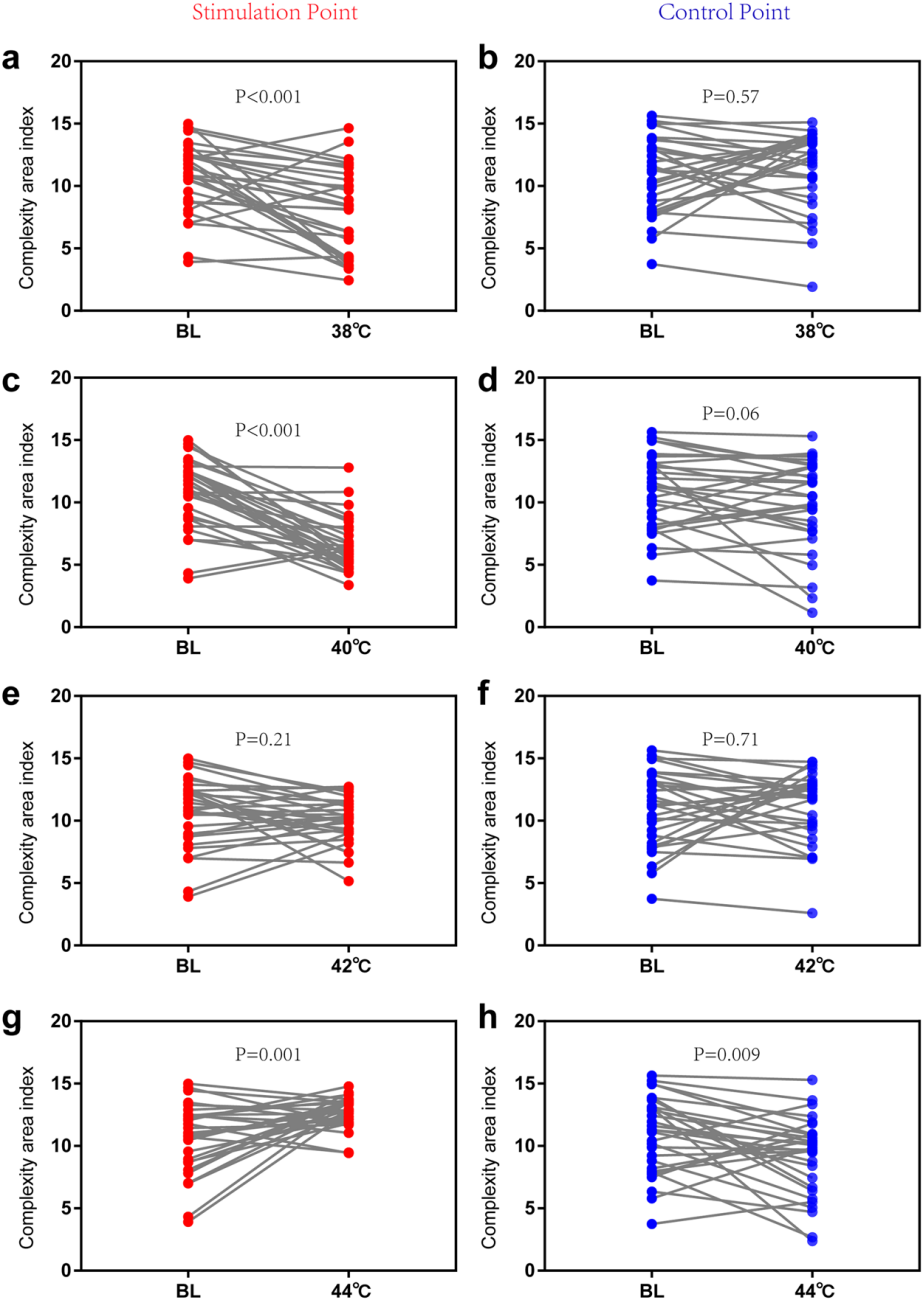
Contrast in MFE between baseline and other thermal stimulation conditions. (**a**) Stimulation point with 38 °C stimulation. (**b**) Control point with 38 °C stimulation. (**c**) Stimulation point with 40 °C stimulation. (**d**) Control point with 40 °C stimulation. (**e**) Stimulation point with 42 °C stimulation. (**f**) Control point with 42 °C stimulation. (**g**) Stimulation point with 44 °C stimulation. (**h**) Control point with 44 °C stimulation. Paired-t test (n = 30). Data are presented as the mean ± SE.

To discriminate the local blood flux pattern following different thermal stimulations, the time series of blood flux signals were analysed using complexity analysis (Fig. 3). To ensure accurate results, two types of complexity analysis methods, multi-scale entropy (MSE) and multi-scale fuzzy entropy (MFE), were conducted in the current study. To evaluate the reliability of the methods, both the MSE and MFE values of white noise were previously calculated, and the result (Fig. S4a) is the same as that previously reported, indicating that our methods and parameters are suitable^21,22^. For blood flux signals, although the value of MFE is less than that of MSE at all scales, the area index of both is always positively linearly correlated under various conditions (Fig. S4b,c,d,e and f). For MSE (Fig. 3a,b and c), there was no significant change with a 38 °C stimulation, while a 40 °C stimulation significantly reduced the area index. As the stimulus temperature increased, the complexity area index also increased. However, after 42 °C stimulation, the complexity area index was still less than that of the control point. When the stimulus temperature increased to 44 °C, the local blood flow complexity showed no significant difference from the control point (Fig. 3c). For MFE (Fig. 3d,e and f), the changes were similar to those of the MSE results (Figs 4 and 5). There were no changes in the complexity in the parallel control group (Fig. S3).

**Figure 6:**
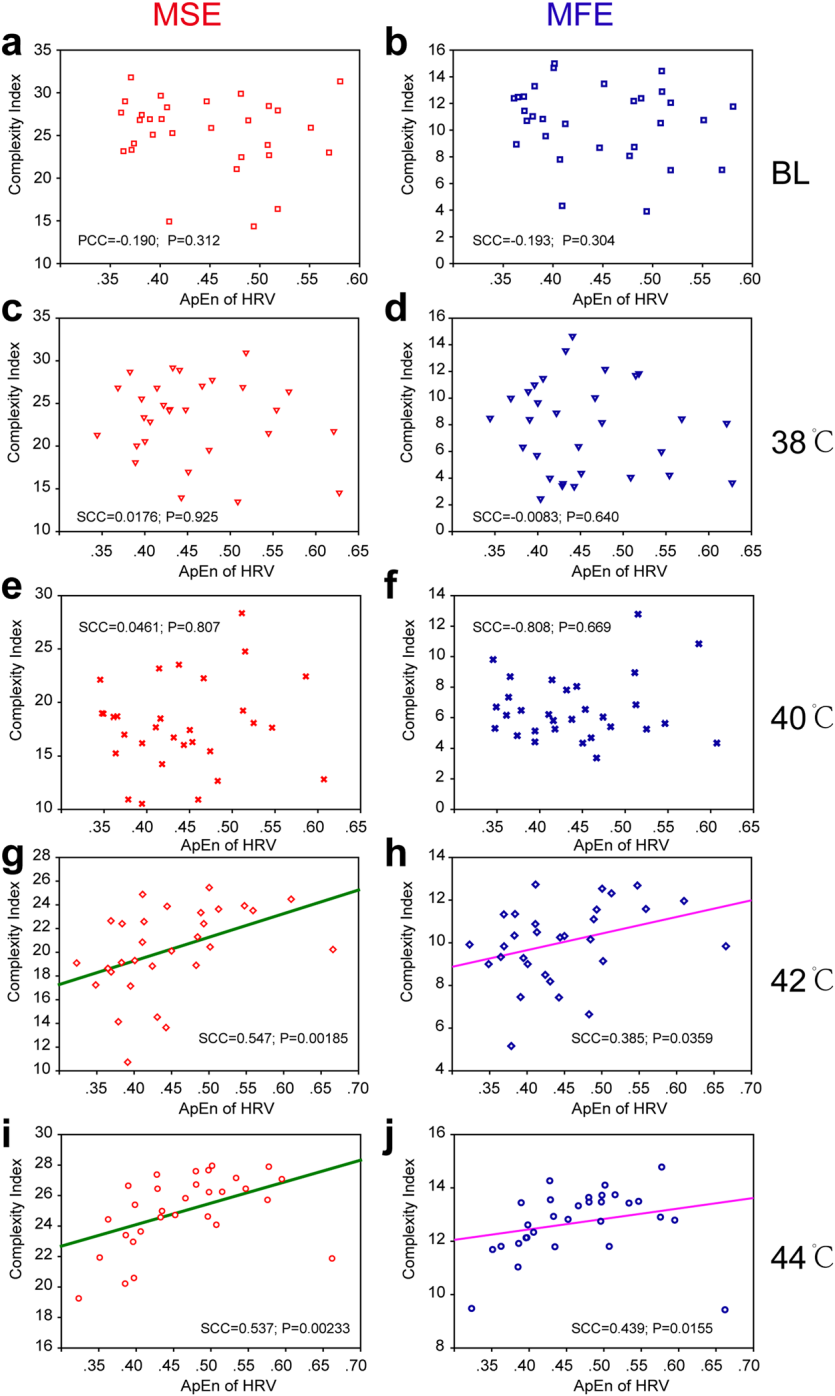
Relationship of the complexity between blood perfusion flux and heart rate variability. (**a**) MSE result at baseline condition. (**b**) MFE result at baseline condition. (**c**) MSE result at 38 °C thermal stimulation. (**d**) MFE result at 38 °C thermal stimulation. (**e**) MSE result at 40 °C thermal stimulation. (**f**) MFE result at 40 °C thermal stimulation. (**g**) MSE result at 42 °C thermal stimulation. (**h**) MFE result at 42 °C thermal stimulation. (**i**) MSE result at 44 °C thermal stimulation. (**j**) MFE result at 44 °C thermal stimulation. SCC, Spearman’s correlation coefficient.

The aforementioned analysis only analysed the complexity of the local blood flux signals. However, an appropriate stimulation not only results in local changes but also leads to systemic reactions. A previous study showed that heart rate and its variability as a comprehensive index can reflect the body’s response to an external stimulus. Approximate entropy (ApEn) is among the indicators of signal complexity^27^. Large values of ApEn indicate high complexity in the signal, and smaller values mean a more regular signal. The results of the current study suggest that different temperature stimuli have no significant effect on the ApEn of heart rate variability (Fig. S5). However, there was a significant linear correlation between local blood flux and heart rate variability at 42 °C and 44 °C of stimulation. This significant linear correlation is not only reflected in the MSE results (Fig. 6g,i) but also in the MFE results (Fig. 6h and j). When the stimulation temperature is less than 42 °C, there is no such correlation (Fig. 6a, b,c,d,e and f), and this correlation also cannot be found in the parallel blank control group (Fig. S6).

## Discussion

The main finding of this study was that the local blood flux signal patterns caused by different temperature stimulations were different. Furthermore, we measured these patterns from the perspective of complexity and then analysed the relationship between the local blood flux and HRV. As far as we know, this study is the first to study the relationship between the local blood flux signal and HRV in terms of complexity and can provide a reference for future research.

Moxibustion is among the most important means of acupuncture treatment and has a long history and extensive application^28^. Although the mechanism of moxibustion is divided into temperature-related action and non-temperature-related action^1^, it is generally believed that the temperature-related action plays essential roles. In the clinic, different temperatures might produce different moxibustion effects largely because of the activation of different subtypes of transient receptor potential (TRP)^29,30^. Because various subtypes of TRP are widely distributed on the surface of mast cells^31,32^, and the activation of mast cells directly regulates local blood flux^33,34^, the impact of different temperature stimulations on local blood flux is necessarily different, and the previous results have provided evidence to support this.

According to previous studies, blood flux oscillations can be separated into different components in the frequency domain^35–38^, which might reflect different physiological rhythms^39^. However, from a complexity perspective point of view, these components are indivisible and are part of a multiscale system. In recent years, complexity analysis, as an effective non-invasive tool, has been used for blood regulation analysis^19,40^. In this study, the complexity of local blood flux signals resulting from different temperature stimulations was different. As the temperature increased, the complexity decreased; however, when the temperature exceeded 40 °C, this trend reversed. Such results suggest that at approximately 40 °C, there had to be a thermal stimulation threshold. Sub-threshold stimulation reduced the complexity and enhanced the certainty of the blood flux signal. However, supra-threshold stimulation increased the complexity of the blood flux regulation and the uncertainty of the blood flux signals. However, the same stimulation did not affect the complexity of the blood flux signal at adjacent points and the complexity of HRV, which suggested that local thresholds and general thresholds are not same. Local supra-threshold stimulation does not result in general global changes, which benefits the maintenance of the stability of the internal environment.

During recent years, studies regarding the moxibustion effect on human HRV have been reported^41,42^, which suggested that heart rate and variability can be used as a specific indicator reflecting thermal stimulating effects on the general body. In this study, the appropriate entropy of heart rate complexity was calculated to evaluate the thermal effect. The results suggest that below 44 °C stimulation, unlike the local blood flux signal, appropriate entropy showed no significant changes in healthy subjects. However, correlation analysis results indicated that when the temperature was higher than 42 °C, the local blood flux signal was positively related to the HRV. In this study, only a narrow range of temperature stimulation was designed and without observed nociceptive heat stimulation. In addition, because the main purpose of this study was to observe the different temperature stimulation results, the effect of the acupoint specificity was not addressed in the current study.

## Conclusion

There were different patterns and complexities of blood perfusion signals following different thermal stimulations. After 42 and 44 °C thermal stimulations, there was a linear correlation between HRV and local blood flux. The current study has two potential values in clinical practice. One is to optimize moxibustion stimulation temperature from the aspect of signal complexity measurement. Secondly, it provides a methodological approach to explore relationship between viscera and acupoints from complexity analysis.

## Methods

### Inclusion and exclusion criteria

Eligible subjects must be healthy and within the age range of 18 to 60 years. All participants were requested to avoid coffee, alcohol and tea for at least 24 hours prior to the measurement. None of the subjects was taking any medication affecting cardiovascular or autonomic regulation.

### Participants and design

A total of 60 healthy subjects were enrolled in the study, and all of the subjects completed measurements and were included in the statistical analysis. The general characteristics are presented in Table 1 and Table S1. In the current study, we set 38 °C and 42 °C stimuli to form experiment 1 and 40 °C and 44 °C stimuli for experiment 2. For each subject, the order of experiment 1 and experiment 2 was determined using a random method. For any experiment, the protocol was as follows (Fig. 1a): All experiments occurred in a quiet, temperature-controlled (24–26 °C) laboratory. After a period of cardiovascular stability (40 min), a baseline recording was made for 30 min (separated into 25 min and 5 min). Then, the test subjects were stimulated by a 38 or 40 °C stimulation point using a PF 5020 unit (Perimed AB, Stockholm, Sweden) for 30 min. At least 30 min after rest, subjects were stimulated by a 40 or 44 °C stimulation at the same point for 30 minutes. Then the subjects remained for a 30-min rest.

### Protocol for measurement of blood perfusion and analysis

The blood perfusion flux signal recording and analysis method is described in our previous studies^43–45^. Both thermal stimulation points and control point were marked as shown in Fig. 1b. Blood perfusion flux signals were recorded using a PeriFlux 5000 (Perimed AB, Stockholm, Sweden) System with a 64-Hz sample rate and a 0.2-s time constant. In this study, a thermal stimulation point was recorded using the first channel of a laser Doppler probe (Probe 457, Perimed AB, Stockholm, Sweden) that combined a laser Doppler and thermostatic probe simultaneously. The control point was recorded using the second channel. The blood flux and temperature records of the case subjects are shown in Fig. 1c. The recorded file for each subject was opened using the software PeriSoft for Windows (version 2.5.5, Perimed, Sweden). The detailed data were exported in txt format and then imported into the Matlab software and analysed. The change ratio of the mean blood flux during every time point was calculated.

### Complexity of blood flux signal

Multiscale entropy (MSE) and multiscale fuzzy entropy were used to measure the complexity of the blood flux signal. The analysis theory is described by Costa^21,22^, and the analytical methods of the Matlab toolbox are provided by physionet^46^. A total of 25 min of blood flux signals was used for the complexity analysis. The analysis parameters used to calculate S_E_ were N = 96000, m = 2, and r = 0.15. Multiscale fuzzy entropy was conducted using the Matlab toolbox^47^. Except for the parameter fuzzy power (in the current study, fuzzy power was equal to 2), the remaining parameters were the as same as those of the MSE analysis. A single index termed the complexity area index was calculated as the area under the multiscale entropy curve^48,49^.

### Electrocardiogram Measurement Protocol and ApEn analysis

The ECG recording and analysis method is described in our previous studies^9–11,43^. The ECG was recorded using the NeurOne system (NeurOne, MEGA electronics Ltd, Finland) at a sampling rate of 1000 Hz. The raw data were exported in an ASC format and then imported into the Kubio HRV software and analysed^50^.

### Statistical analysis

Data are expressed as the mean ± SE. A paired-t test analysis was used to assess the difference between different points and different temperatures. The correlation between the complexity area index and HRV ApEn was analysed using Spearman’s correlation coefficient (SCC). All tests and statistical analyses were conducted using SPSS software (Version 13.0, SPSS Inc., Chicago, IL). The level of significance was defined as *P* < 0.05. All reported P values were two-sided.

### Ethics approval and consent to participate

This study was approved by the Institutional Research Ethics Boards of Acupuncture & Moxibustion, China Academy of Chinese Medical Sciences. In accordance with the Declaration of Helsinki, each subject provided informed consent and had an adequate understanding of the procedure and purpose of the study.

## Electronic supplementary material

Supplementary materials
