# Author Correction: Exploring the Relationship between Blood Flux Signals and HRV following Different Thermal Stimulations using Complexity Analysis

**DOI:** 10.1038/s41598-018-29003-7

**Published:** 2018-07-11

**Authors:** Guangjun Wang, Shuyong Jia, Hongyan Li, Ze Wang, Weibo Zhang

**Affiliations:** Institute of Acupuncture and Moxibustion, China Academy of Chinese Medical Sciences, Beijing, China

Correction to: *Scientific Reports* 10.1038/s41598-018-27374-5, published online 12 June 2018

In the Supplementary Materials file originally published with this Article, the Article title was given incorrectly. This error has been corrected in the Supplementary Materials that now accompanies the Article.

