# Supplementary material for: Exploring the Relationship between Blood Flux Signals and HRV following Different Thermal Stimulations using Complexity Analysis
Source: Sci Rep. 2018 Jun 12;8:8982. doi: 10.1038/s41598-018-27374-5 (PMC5997638; doi:10.1038/s41598-018-27374-5)
Supplement: Supplementary file 1 — Supplementary materials [file 41598_2018_27374_MOESM1_ESM.docx]

**Exploring the Relationship between Blood Flux Signals and HRV following Different Thermal Stimulations using Complexity Analysis**

Guangjun Wang*, Shuyong Jia, Hongyan Li, Ze Wang, Weibo Zhang

Institute of Acupuncture and Moxibustion, China Academy of Chinese Medical Sciences, Beijing, China

Guangjun Wang: tjuwgj@gmail.com

Shuyong Jia: shuyong6666@163.com

Hongyan Li: lhylhyz90@163.com

Ze Wang: 412272354@qq.com

Weibo Zhang [zhangweibo@hotmail.com](mailto:zhangweibo@hotmail.com)

| Table S1. Subject's characters description of blank control group (9M/21F) | | | |
| --- | --- | --- | --- |
|  | Min | Max | Mean±SD |
| Age (years) | 21 | 31 | 25±2 |
| Height (cm) | 155 | 180 | 165.77±6.54 |
| Weight (kg) | 47 | 95 | 60.13±9.52 |
| BMI | 17.87 | 30.32 | 21.81±2.53 |


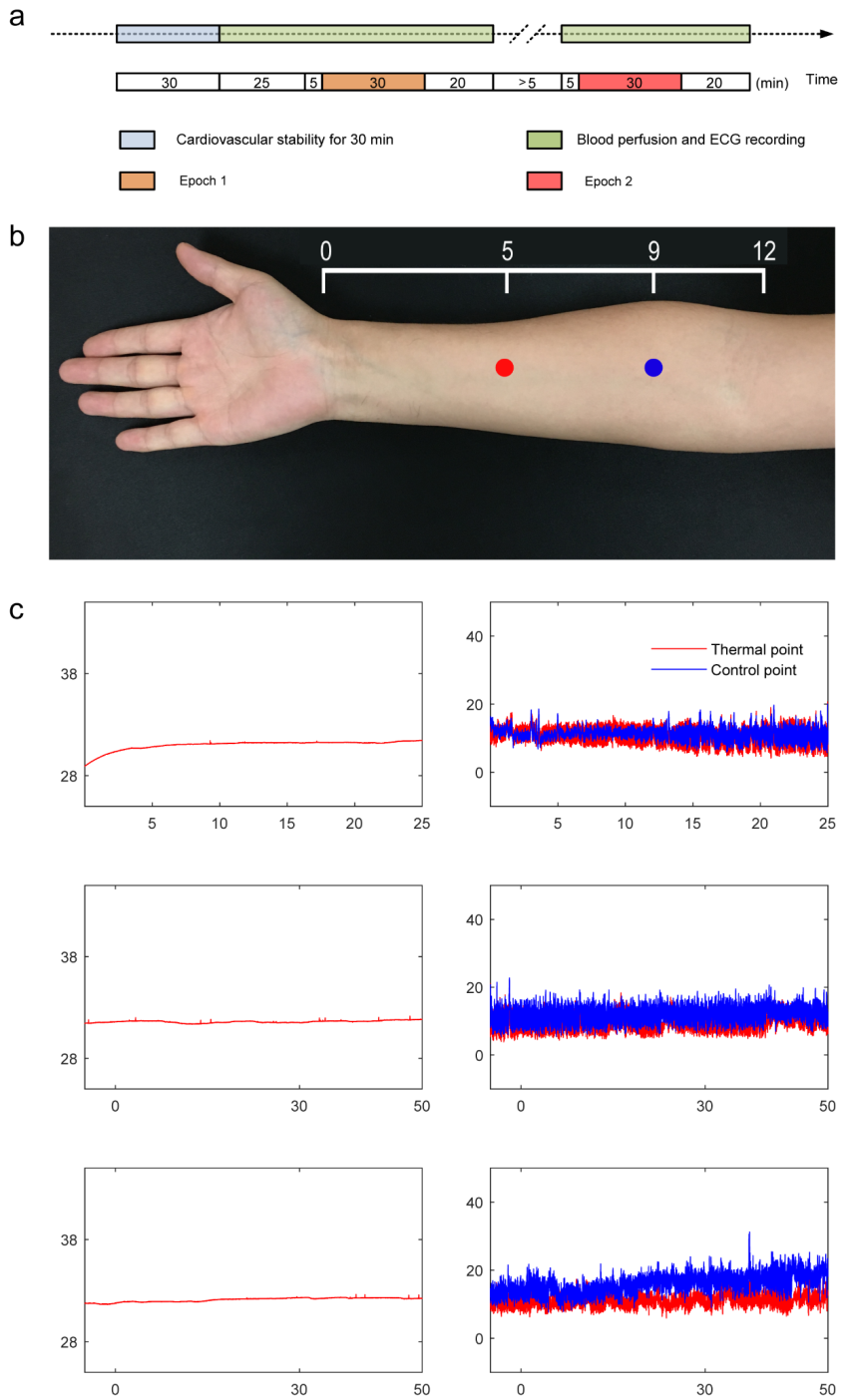


Figure S1. Experiment design and case subject’s raw data of blank control group. (a) Experiment design in blank control group without thermal stimulation. (b) Thermal stimulation point(red) and control point(blue) location of the right forearm. The thermal stimulation point is located on the anterior aspect of the forearm, between the tendons of the palmaris longus and the flexor carpi radials, 5 B-cun proximal to the palmar wrist crease. The control point is 7 B-cun proximal to the palmar wrist creas. (c) Temperature of thermal stimulation point(left) and the blood perfusion signals both on thermal stimulation and control points(right).


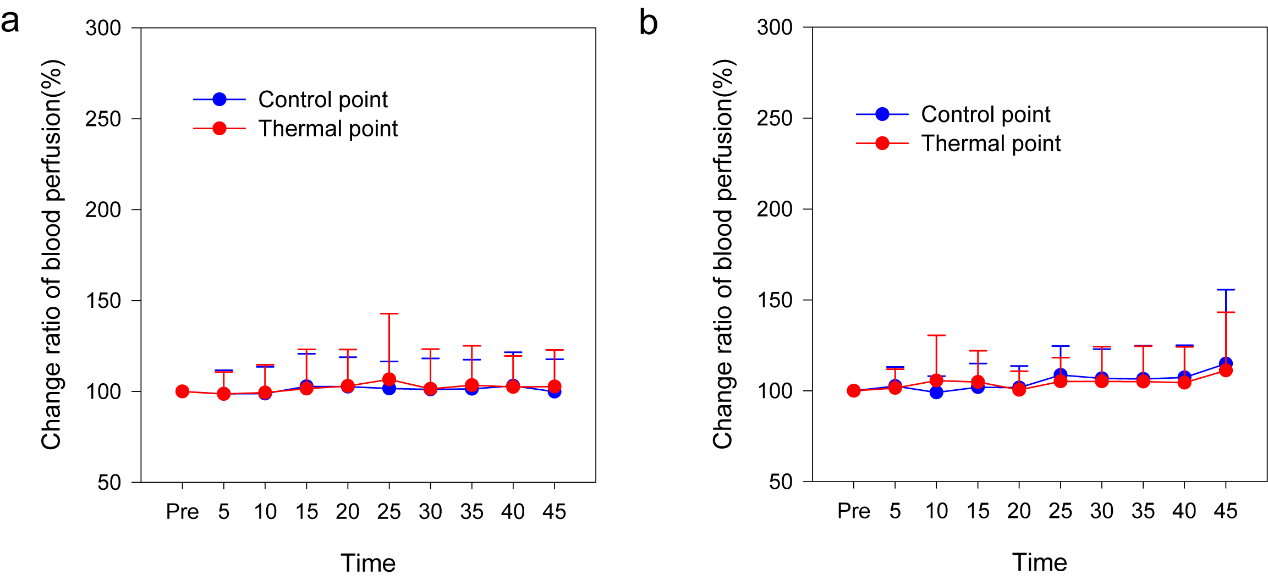


Figure S2. Blood perfusion flux signals both on the stimulation and control points. (a) first epoch. (b) second epoch. Data presented as Mean±SD. P>0.05, paired-t test(n=30).


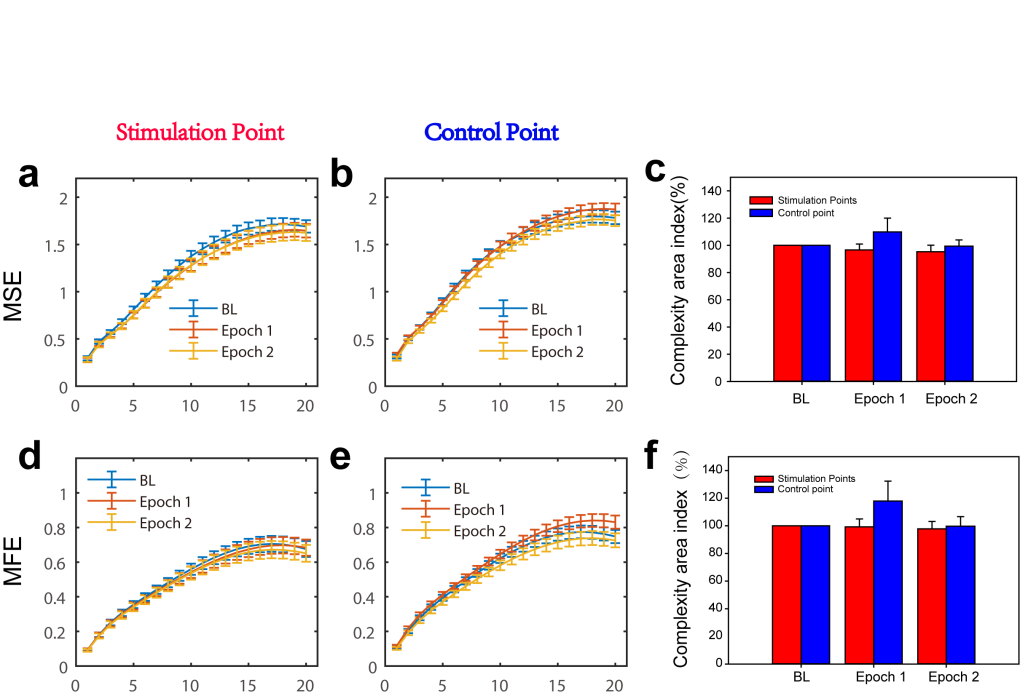


Figure S3. Complexity of blood perfusion flux signals in parallel blank control group. (a) MSE result of different scales on stimulation point. (b) MSE result of different scales on control point. (c) Complexity area index of different epochs and different points. Complexity area index was obtained as the area under the multiscale entropy curve of Fig. 3a and Fig. 3b. (d) MFE result of different scales on stimulation point. (e) MFE result of different scales on control point. (f) Complexity area index of different conditions and different points. Complexity area index was obtained as the area under the multi-scale entropy curve of Fig. 3d and Fig. 3e. P>0.05, paired-t test(n=30). MSE, multi scales entropy. MFE, multiscale fuzzy entropy. BL, baseline condition without stimulation. Data presented as Mean±SE.


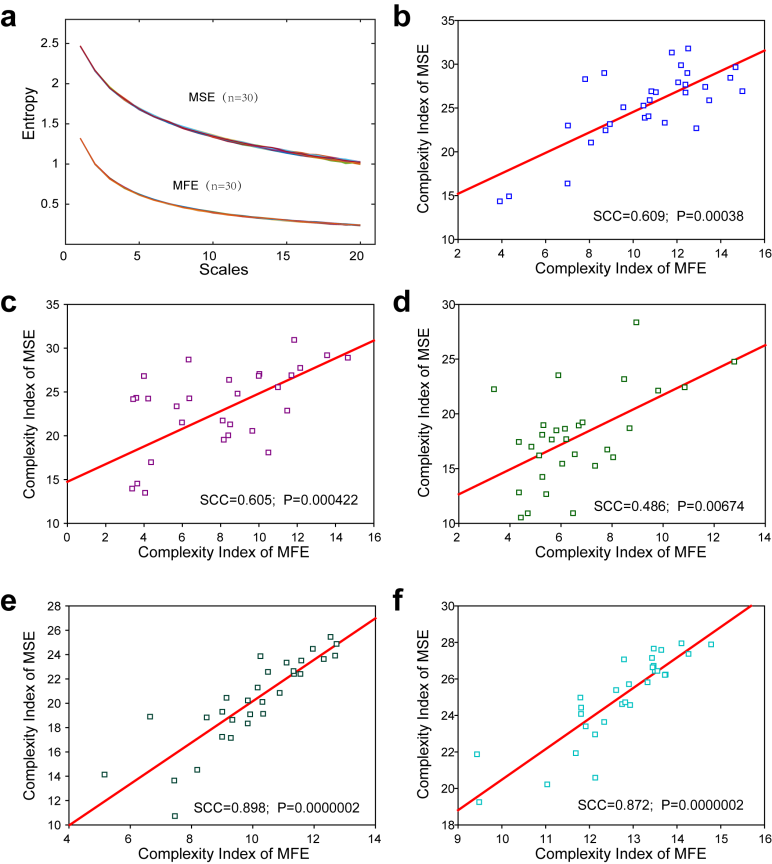


Figure S4. Correlation analysis of MSE and MFE. (a) MSE and MFE value of white noise signals. Both the values ​​of MSE and MFE are derived from white noise signals with a data length of 96000. (b) Correlation at baseline condition. (c) Correlation at 38℃ stimulation. (d) Correlation at 40℃ stimulation. (e) Correlation at 42℃ stimulation. (d) Correlation at 44℃ stimulation. SCC, Spearman’s Correlation Coefficient.


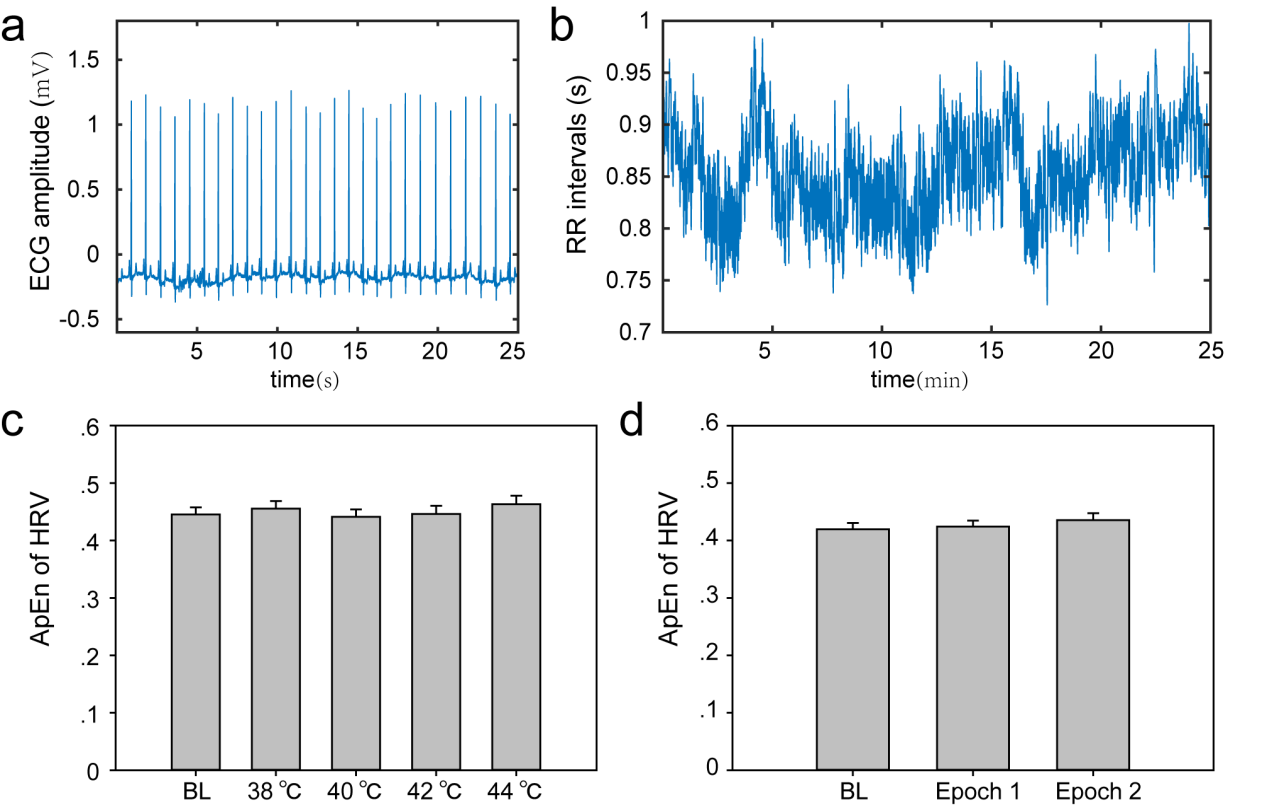


Figure S5. Complexity of HRV. (a) Raw data of ECG. (b) RR intervals of ECG. (c) Complexity of HRV under different thermal stimulations. (d) Complexity of HRV in parallel blank control group. P>0.05. Data presented as Mean±SE.


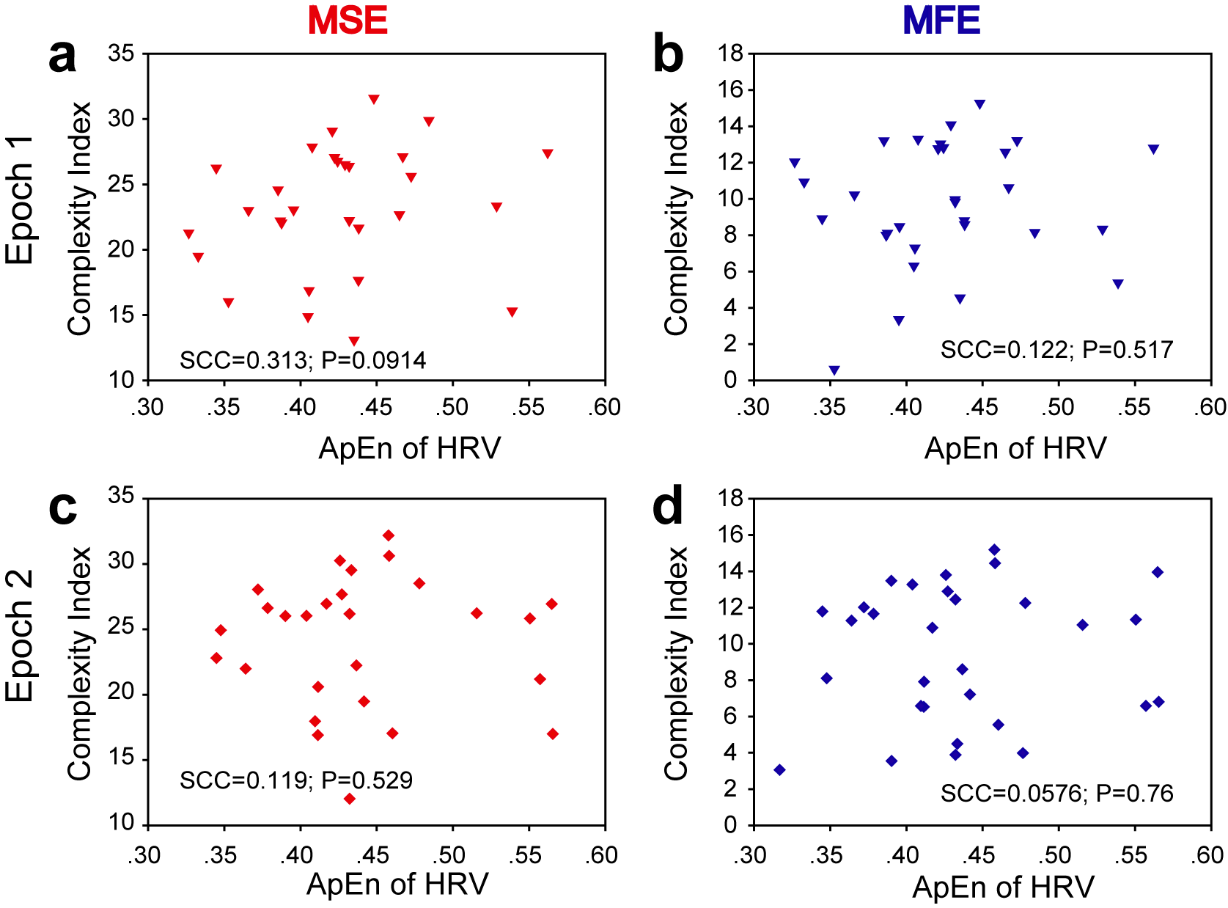


Figure S6. Relationship of complexity between blood perfusion flux heart rate variability in parallel blank control group. (a) MSE result at epoch 1. (b) MFE result at epoch 1. (c) MSE result at epoch 2. (d) MFE result at epoch 2. SCC, Spearman’s correlation coefficient.
